# Interpreting predictive performance in AI-based CCTA adipose-tissue models: from model evaluation to clinical transportability

**DOI:** 10.1093/ehjdh/ztag174

**Published:** 2026-10-30

**Authors:** Sina Kazemian, Zahra Vaziri, Kaveh Hosseini

**Affiliations:** Tehran Heart Center, Cardiovascular Diseases Research Institute, Tehran University of Medical Sciences, Tehran, Iran; Tehran Heart Center, Cardiovascular Diseases Research Institute, Tehran University of Medical Sciences, Tehran, Iran; Department of Cardiology, School of Medicine, Babol University of Medical Sciences, Babol, Iran; Department of Cardiology, Copenhagen University Hospital-Herlev and Gentofte, Gentofte Hospitalsvej 1, 2900 Hellerup, Copenhagen, Denmark

We read with great interest the systematic review and meta-analysis by Salehi *et al*. examining artificial intelligence (AI)-based models incorporating coronary computed tomography angiography (CCTA)-derived adipose-tissue characteristics for cardiovascular risk prediction.^1^ The authors provide a timely synthesis of an emerging field and acknowledge important limitations, including methodological heterogeneity, limited external validation, imaging variability, potential overfitting and data leakage, and concerns regarding generalizability. Building on this discussion, we examine clinical comparability and within-dataset dependence among area under the receiver operating characteristic curve (AUC) estimates (*Figure 1*).

**Figure 1:**
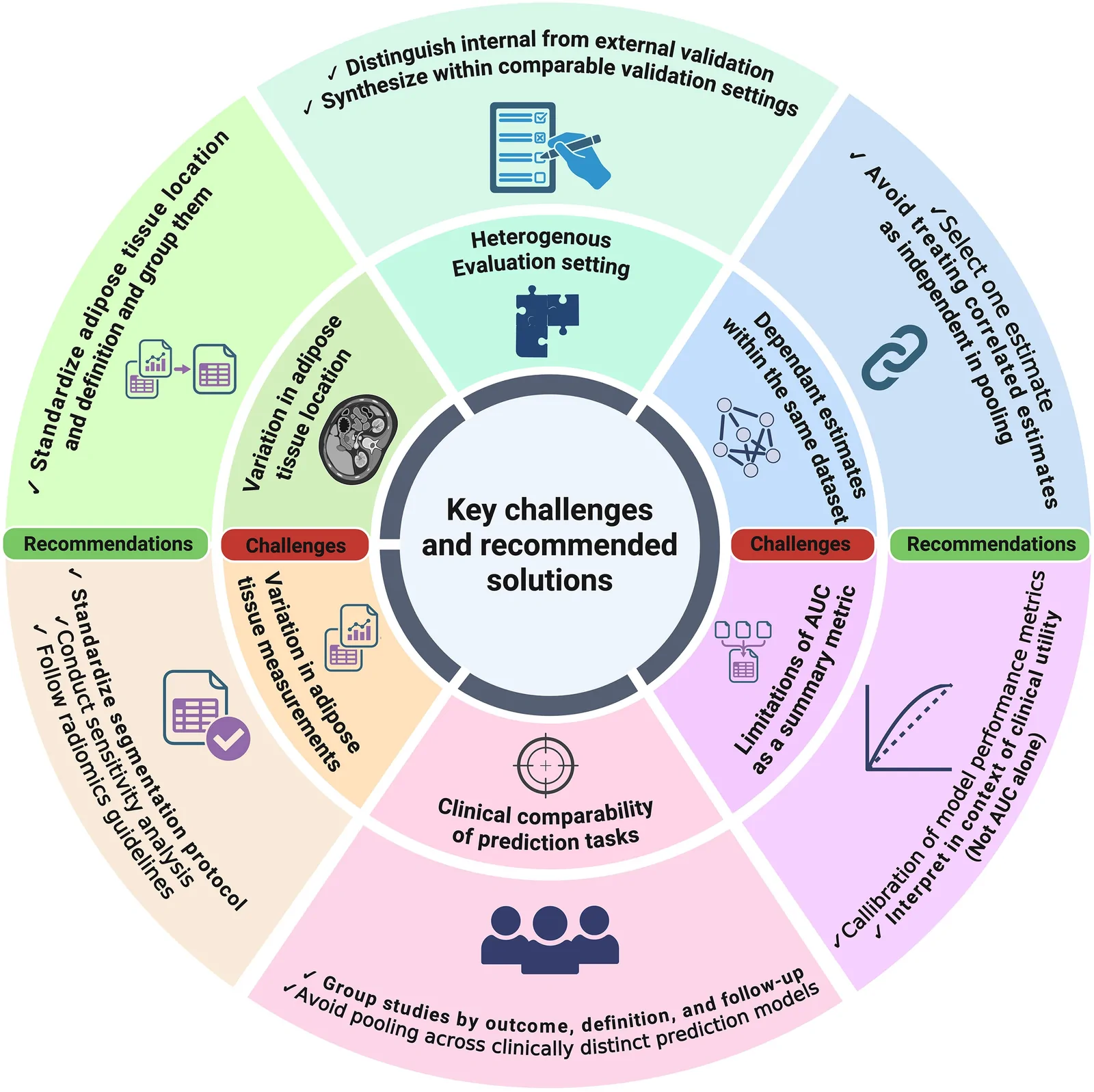
Key methodological challenges and recommended solutions for meta-analysis of AI-based CCTA adipose-tissue prediction models. The figure highlights major challenges related to validation setting, within-dataset dependence, AUC interpretation, clinical comparability, and adipose-tissue measurement, together with corresponding recommendations for more robust evidence synthesis. AI, artificial intelligence; AUC, area under the receiver operating characteristic curve; CCTA, coronary computed tomography angiography.

## Interpretation of pooled model performance

The authors defined each algorithm-feature set-dataset split as a distinct model evaluation. Their sensitivity analyses yielded a pooled AUC of 0.819 across 74 model evaluations and 0.836 when restricted to the best-performing evaluation per study, preferentially selecting test-set or external-validation estimates when available.^1^ As acknowledged by the authors, only three studies reported external validation in separate cohorts. Unlike internal cross-validation, external validation assesses performance outside the model-development dataset and more directly informs transportability.^2,3^ Combining these validation settings therefore warrants caution when interpreting pooled AUCs as evidence of clinical readiness.

The all-model synthesis included potentially correlated AUC estimates from evaluations involving overlapping participants or the same datasets. Although the authors did not describe these evaluations as independent studies, including them as separate effects in a conventional inverse-variance random-effects meta-analysis without accounting for their covariance may give greater influence to datasets contributing more model evaluations and may affect the estimated standard error and confidence interval of the pooled AUC.^4^ In particular, Chan *et al*. included a large cohort of 43 884 participants and contributed two training-set AUC estimates to the all-model synthesis. In the leave-one-model-evaluation-out analysis, individually omitting these estimates produced the largest changes in the pooled AUC (from 0.819 to 0.802 and 0.803, respectively).^1^ These findings indicate influence at the individual-evaluation level, whereas the combined dataset-level influence and implications of within-dataset covariance remain undetermined.

## Interpretation of pooled AUCs

A related consideration concerns the interpretation of meta-analytic weighting. Inverse-variance random-effects weights depend on within-evaluation and between-effect variance, not directly on model quality or transportability. AUC is a bounded, rank-based measure that varies with case mix and prediction task. Although AUCs can be pooled, the random-effects estimate represents the mean of an assumed distribution of evaluation-specific AUCs, not necessarily the performance of a particular model in a defined setting. Accordingly, clinical interpretation depends on comparable populations, prediction tasks, predictors, and validation settings.^4^

Future syntheses could prespecify the performance estimand and separately summarize internal and external validation when sufficient evidence is available. When multiple model evaluations arise from the same dataset, dependence may be addressed through prespecified selection of a single relevant estimate or, when sufficient independent datasets and within-dataset covariance information are available, appropriate multilevel or multivariate methods that account for within-dataset covariance. Where prediction tasks or validation settings remain clinically or methodologically distinct, separate or stratified synthesis may be more informative than a single pooled AUC.^2,5^

## Clinical comparability of the prediction tasks

Beyond the variation in major adverse cardiovascular event (MACE) definitions and follow-up acknowledged by the authors, included studies addressed distinct tasks. Miao *et al*. and Militello *et al*. evaluated coronary artery disease classification; Huang *et al*. predicted future acute coronary syndrome (ACS), Wei He *et al*. predicted percutaneous coronary intervention, and others predicted future MACE.^1,6–9^ Classification concerns disease status at assessment, whereas prognostic models predict subsequent events. Revascularization also depends partly on subsequent clinical and treatment decisions.

Consequently, the pooled AUCs from both analyses are best interpreted as overall summaries of discrimination across heterogeneous prediction tasks rather than estimates of performance for a specific clinical outcome. Likewise, prognostic models evaluated over different prediction horizons may not represent directly comparable prediction targets. Future syntheses could prespecify the clinical task and prediction horizon, distinguish diagnostic from prognostic models, and consider task-specific quantitative synthesis when sufficient comparable evaluations are available.

AUC may also differ across populations with differing baseline cardiovascular risk or established disease. Likewise, models based on different adipose-tissue depots, including epicardial and perivascular adipose tissue, and differently defined imaging features such as fat attenuation index and radiomics, should not be assumed to be directly interchangeable. These differences concern clinical comparability, not only statistical heterogeneity.^3,10^

## Robustness and algorithm comparisons

The dependence structure also affects interpretation of the leave-one-out analysis. In the all-model synthesis, one AUC estimate was omitted at each iteration, while other estimates from the same dataset could remain. The reported leave-one-effect-size-out AUC range (0.802–0.824) supports the robustness of the pooled estimate to omission of individual model evaluations but does not establish robustness to removal of an entire underlying dataset or quantify the effect of within-dataset dependence on the reported uncertainty.^5^ The authors also reported a leave-one-out influence analysis for the best-model subset (AUC range: 0.809–0.840), which they described as leave-one-study-out. To further strengthen the findings, a leave-one-dataset-out sensitivity analysis could assess dataset-level influence, while a dependence-aware meta-analysis could assess the implications of within-dataset covariance for the pooled AUC and its confidence interval.

A similar consideration applies to algorithm-class comparisons. The all-model analysis reported a statistically significant overall difference across algorithm classes (*P* < 0.001), with the highest pooled AUC observed for deep learning, whereas the best-model comparison did not reach conventional statistical significance (*P*: 0.061).^1^ Huang *et al*. was the only included deep-learning study, comprising 100 patients and contributing six convolutional neural network model evaluations from the same dataset.^9^ Together, these findings highlight the limited independent evidence available to support conclusions regarding the superiority of deep learning across populations, as repeated model evaluations within a single dataset provide less evidence of generalizability than comparable findings replicated across independent cohorts. Further head-to-head comparisons using common datasets and consistent validation procedures, together with replication across independent patient cohorts, are needed to better understand the relative predictive performance and generalizability of different algorithm classes.

In conclusion, Salehi *et al*. provide a valuable synthesis of AI-enabled CCTA adipose-tissue analysis.^1^ Greater attention to the target performance estimand, clinical prediction task, validation setting, and independence of the underlying datasets would further strengthen interpretation of the quantitative evidence. A validation- and dataset-aware approach may help distinguish promising discrimination from reproducible and transportable predictive performance in clinical settings.

## Data Availability

The data underlying this article are available in the article.
